# Pan-cancer analyses reveal IGSF10 as an immunological and prognostic biomarker

**DOI:** 10.3389/fgene.2022.1032382

**Published:** 2023-01-04

**Authors:** Yongxia Zhou, Manzhi Gao, Yaoyao Jing, Xiaofang Wang

**Affiliations:** ^1^ Department of Hematology, Tianjin Medical University Cancer Institute & Hospital, Tianjin, China; ^2^ Tianjin Medical University Cancer Institute & Hospital, National Clinical Research Center for Cancer, Tianjin, China; ^3^ Tianjin’s Clinical Research Center for Cancer, Tianjin, China; ^4^ Key Laboratory of Breast Cancer Prevention and Therapy, Tianjin Medical University, Ministry of Education, Tianjin, China; ^5^ Key Laboratory of Cancer Immunology and Biotherapy, Tianjin, China; ^6^ Day Ward of Tianjin Medical University Cancer Institute & Hospital, Tianjin, China

**Keywords:** *IGSF10*, pan-cancer, prognosis, immune infiltration, TMB, MSI, drug sensitivity

## Abstract

**Background:** IGSF10 is a member of the immunoglobulin superfamily. Over the previous decade, growing proof has validated definitive correlations between individuals of the immunoglobulin superfamily and human diseases. However, the function of *IGSF10* in pan-cancer stays unclear. We aimed to analyze the immunological and prognostic value of *IGSF10* in pan-cancer.

**Methods:** We utilized a vary of bioinformatic ways to inspect the function of *IGSF10* in pan-cancer, including its correlation with prognosis, immune cell infiltration, tumor mutational burden (TMB), microsatellite instability (MSI), mismatch repair (MMR), DNA methyltransferases, genetic alteration, drug sensitivity, etc.

**Results:** We noticed low expression of *IGSF10* in most cancer types. *IGSF10* expression in tumor samples correlates with prognosis in most cancers. In most cancer types, *IGSF10* expression was strongly related to immune cells infiltration, immune checkpoints, immune modulators, TMB, MSI, MMR, and DNA methyltransferases, among others. Functional enrichment analyses indicated that *IGSF10* expression was involved in lymphocyte differentiation, cell molecules adhesion, etc. Furthermore, low *IGSF10* expression could increase the drug sensitivity of many drugs.

**Conclusion:** *IGSF10* could serve as a novel prognostic marker and attainable immunotherapy target for several malignancies.

## 1 Introduction

Cancer has grown to be one of the principal causes of human death in every country around the world and a vital obstacle for countries in the world to increase human life expectancy (Sung et al., 2021). In 2020, greater than 19 million humans have been recognized with cancer, and almost 10 million human beings have died from cancer in countries around the world. Some researchers predict that by 2040, there will be about 28 million new instances of cancer and 16 million deaths due to cancer, respectively (Mao et al., 2022). Molecular profiling of tumor tissues from patients in various cancer types has been notably and intensively studied over the previous few years, and these studies have driven rapid advances in “personalized” or “precision” medicine (El-Deiry et al., 2019). As the attentions and efforts in precision care increase, there is a recognition not only of the importance of biomarkers, but also how they can be used for targeted therapies in clinical research is critical (Park et al., 2020).

Immunoglobulin superfamily (IgSF) proteins include many members, which are widely present in most types of cells, and this family of proteins is a class of cell surface proteins with multiple functions (Wojtowicz et al., 2020). IGSF10 is a member of IGSF with multiple biological functions, and its gene mutation affects the migration of gonadotropin-releasing hormone neuronal, which can lead to delayed puberty (Howard et al., 2016). To date, little has been reported about the role of IGSF10 in tumors. Previous study reported that significantly reduced expression of *IGSF10* was detected in a radiation-induced rat osteosarcoma model (Daino et al., 2009). A recent study investigated that *IGSF10* expression was considerably reduced in lung cancer, and knockdown of *IGSF10* promoted malignant progression of lung cancer cells (Ling et al., 2020). Furthermore, another research determined that the expression of *IGSF10* was down-regulated in breast cancer tissues, and *IGSF10* expression was related to good prognosis (Wu et al., 2021). Although researchers have conducted multiple studies on *IGSF10*, until now, the role of *IGSF10* in most cancers has remained largely unknown.

To this end, our research is the first to perform a pan-cancer analysis of *IGSF10* based on multiple databases to comprehensively understand the function of *IGSF10* in pan-cancer. We analyzed the *IGSF10* expression in different tumor tissues and normal tissues. We further investigated the prognostic value of *IGSF10* and the correlations between *IGSF10* and immune cells infiltration, immune-related genes, TMB, MSI, MMR, and drug sensitivity. Furthermore, we performed functional enrichment analysis of *IGSF10*-related genes to expose the potential molecular pathogenesis of various cancers. Taken together, the results of our study suggest that *IGSF10* can act as a novel prognostic marker and potential immunotherapy target for several malignancies.

## 2 Materials and methods

### 2.1 Data processing and differential expression analysis

Firstly, we downloaded gene expression information of different normal tissues from the GTEx database. We obtained gene expression information for various tumor cell lines in 29 tissues from the CCLE database. Gene expression information from 33 different tumor tissues in the TCGA database were analyzed using online software UALCAN. We obtained the RNA-seq information of different tumor and paired normal specimens of TCGA dataset uniformly processed by the UCSC XENA database through the Xian Tao academic tools (https:// www.xiantao.love/). We also obtained the RNA-seq information of different tumor and normal specimens of TCGA and GTEx datasets uniformly processed by the UCSC XENA database through the Xian Tao academic tools. RNA-seq information in TPM format were log2 transformed and then analyzed. Data analysis was performed using R software (version 3.6.3), and the R package “ggplot2 (version 3.3.3)” was utilized for visualization. We analyzed the expression differences of *IGSF10* gene in pan-cancer tissues of different cancer stages and normal tissues by using the online software UALCAN.

### 2.2 Survival prognosis analysis

We acquired RNA-seq information from the TCGA database through Xian Tao academic tools. RNA-seq information in FPKM format was transformed to TPM format and log2 transformation was conducted, while retaining samples with clinical information. We selected overall survival (OS), disease-specific survival (DSS) and progression-free interval (PFI) to examine the relationships between *IGSF10* expression and prognostic information in cancer patients. Survival analyses were performed for patients with different tumor types by utilizing univariate Cox regression analysis. Data were analyzed by utilizing R software (version 3.6.3), R package “survival (version 3.2–10)” was utilized for statistical analysis of survival data, and R package “survminer (version 0.4.9)” was utilized for visualization. Furthermore, we additionally analyzed the relationships between *IGSF10* expression and OS in a vary of cancers using Kaplan-Meier online tool. Long-term Outcome and Gene Expression Profiling Database of pan-cancers (LOGpc) was used to study the relationships between *IGSF10* expression and prognosis in GSE13507, GSE31684, GSE20685, GSE31448, GSE31210, GSE3141, GSE41271, GSE62254, GSE29623, and GSE40967.

### 2.3 Immune-related analysis

We obtained data on the correlations between expression of *IGSF10* and immune cell infiltration in pan-cancer from the TCGA database through Assistant for Clinical Bioinformatics platform. The R software package “immunedeconv” and the TIMER algorithm were used to estimate immune cell infiltration levels. The expression data of eight common immune checkpoint-related genes have been extracted, and correlations between *IGSF10* expression and the expression of immune checkpoint-related genes have been noticed. R software program (version 4.0.3) was utilized to operate statistical analysis on obtained data. RNA-seq information for different tumors from the TCGA database have been downloaded through the Xian Tao academic tools. The stromal score, immune score and ESTIMATE score of multiple cancers have been obtained *via* R package “estimate (version 1.0.13)”.

In addition, we downloaded data on correlations between expression of *IGSF10* and expression of immune-related genes from the TIMER 2.0 online website, particularly involving genes encoding immune stimulators, immune inhibitors, and major histocompatibility complex (MHC) molecules. The visualization results have been presented *via* R package “ggplot2 (version 3.3.3)”.

Finally, we downloaded data on correlations between expression of *IGSF10* and TMB, MSI through Assistant for Clinical Bioinformatics platform. The correlations between expression of *IGSF10* and TMB, MSI have been assessed by Spearman correlation analysis. The visualization results were presented by the R package “ggradar (version 0.2)” and “ggplot2 (version 3.3.3)”.

### 2.4 DNA MMR genes and methyltransferases analysis

We downloaded the information on the correlations between *IGSF10* expression and five DNA repair genes (*MLH1*, *MSH2*, *MSH6*, *PMS2*, *EPCAM*), four methyltransferases genes (*DNMT1*, *DNMT3L*, *DNMT3A*, *DNMT3B*) from the TIMER 2.0 online website. The correlations between *IGSF10* expression and five DNA repair genes, four methyltransferases genes were assessed by way of Spearman correlation analysis. The visualization outcomes have been presented by R package “ggplot2 (version 3.3.3)”.

### 2.5 Genetic alteration analysis

GSCA is an integrated platform, which integrates over 10,000 multi-dimensional genomic information across 33 kinds of tumors from TCGA. We used the GSCA online platform to analyze *IGSF10* mutations in different cancers, such as Single Nucleotide Variation (SNV), Copy Number Variation (CNV), and Methylation. Moreover, we also analyzed the relationships between *IGSF10* gene alterations and prognosis in different tumors using the GSCA.

### 2.6 Drug sensitivity analysis

We performed drug sensitivity analysis using the GSCA database, which integrated the gene expression profile and drug sensitivity information in Genomics of Drug Sensitivity in Cancer (GDSC) and The Cancer Therapeutics Response Portal (CTRP) for investigation. The correlations between *IGSF10* expression and drug IC50 were assessed by Pearson correlation analysis.

### 2.7 *IGSF10*-related gene enrichment analysis

We used the GeneMANIA database to analyze IGSF10 interacting proteins and obtained 20 IGSF10-binding proteins. Furthermore, we obtained the top 1000 *IGSF10*-related genes based on TCGA data through the GEPIA2 database. We utilized GEPIA2 to examine correlations between *IGSF10* expression and the top five *IGSF10*-related genes, and correlations between *IGSF10* expression and the top five *IGSF10*-related genes was assessed by Pearson correlation analysis. Two datasets were combined to operate GO enrichment and KEGG pathway analysis. The R package “clusterProfiler (version 3.14.3)” was utilized to operate GO enrichment and KEGG pathway analysis. The R package “org.Hs.eg.db (version 3.10.0)” was utilized to operate ID conversion.

## 3 Results

### 3.1 *IGSF10* is differentially expressed in normal and tumor tissues

**FIGURE 1:**
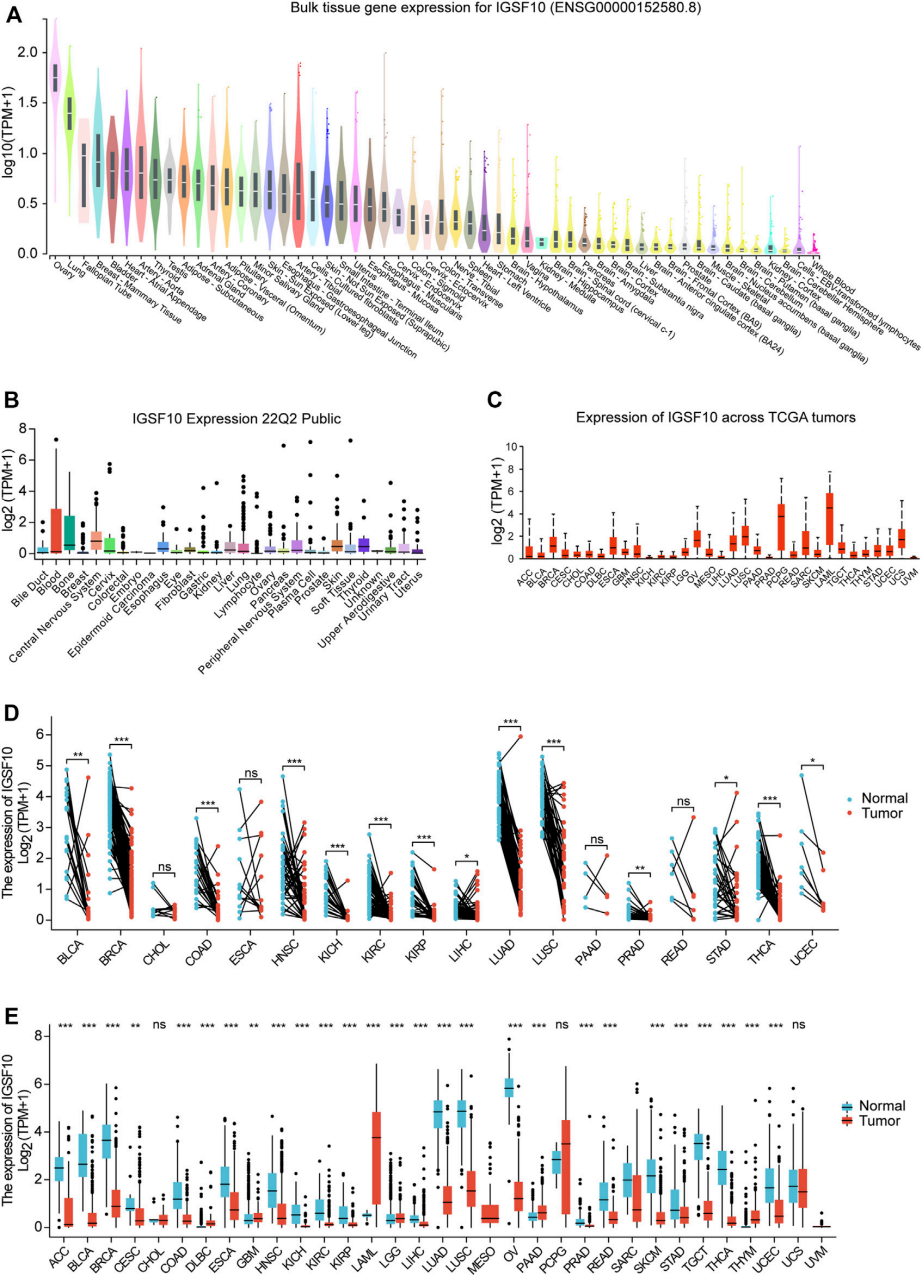
Differential expression of *IGSF10*. **(A)** Expression of *IGSF10* in normal specimens. **(B)** Expression of *IGSF10* in a vary of cancer cell lines. **(C)** Expression of *IGSF10* in 33 kinds of cancer. **(D)** Comparison of *IGSF10* expression between tumor and paired normal specimens. **(E)** Comparison of *IGSF10* expression between tumor and normal specimens. ns, *p* ≥ 0.05, *p < 0.05, **p < 0.01, ***p < 0.001.

To elucidate the physiological expression of *IGSF10* in normal specimens, we explored expression levels of *IGSF10* in different normal specimens using the GTEx database and ranked them from high to low. The results showed that *IGSF10* expression was highest in ovarian tissue, and the lowest in whole blood (Figure 1A). Subsequently, we obtained and analyzed expression data for every tumor cell line from the CCLE database, and there have been variations in expression amongst the cell lines for 29 tumors (Figure 1B). Next, we analyzed *IGSF10* expression in a range of tumor tissues from the TCGA database. *IGSF10* was expressed differently in 33 tumor tissues, with the highest expression in Acute myeloid leukemia (LAML) (Figure 1C). Furthermore, we analyzed and compared the *IGSF10* expression levels between different tumors and paired normal specimens. Significant variations in *IGSF10* expression between tumors and paired normal specimens were detected in 14 tumors, except for these tumors without paired normal specimen data. Most tumor tissues had lower *IGSF10* expression than paired normal tissues, such as Bladder urothelial carcinoma (BLCA), Breast invasive carcinoma (BRCA), Colon adenocarcinoma (COAD), Head and neck squamous cell carcinoma (HNSC), Kidney chromophobe (KICH), Kidney renal clear cell carcinoma (KIRC), Kidney renal papillary cell carcinoma (KIRP), Liver hepatocellular carcinoma (LIHC), Lung adenocarcinoma (LUAD), Lung squamous cell carcinoma (LUSC), Prostate adenocarcinoma (PRAD), Stomach adenocarcinoma (STAD), Thyroid carcinoma (THCA) and Uterine corpus endometrial carcinoma (UCEC). The Cholangiocarcinoma (CHOL), Esophageal carcinoma (ESCA), Pancreatic adenocarcinoma (PAAD) and Rectum adenocarcinoma (READ) cohorts had similar *IGSF10* expression in contrast to paired normal specimens (Figure 1D).

Finally, we integrated RNA-seq information to analyze the *IGSF10* expression in a vary of cancer and normal samples (Figure 1E). Thereinto, *IGSF10* levels were downregulated in Adrenocortical carcinoma (ACC), BLCA, BRCA, Cervical squamous cell carcinoma and endocervical adenocarcinoma (CESC), COAD, ESCA, HNSC, KICH, KIRC, KIRP, LIHC, LUAD, LUSC, Ovarian serous cystadenocarcinoma (OV), PRAD, READ, Skin cutaneous melanoma (SKCM), STAD, Testicular germ cell tumors (TGCT), THCA, and UCEC. In contrast, *IGSF10* had higher expression levels in tumors relative to normal samples in Lymphoid neoplasm diffuse large B-cell lymphoma (DLBC), Glioblastoma multiforme (GBM), LAML, Brain lower grade glioma (LGG), PAAD and Thymoma (THYM). However, there was no notable change in the expression level of IGSF10 between CHOL, Pheochromocytoma and paraganglioma (PCPG), Uterine carcinosarcoma (UCS), and non-tumor tissues.

**FIGURE 2:**
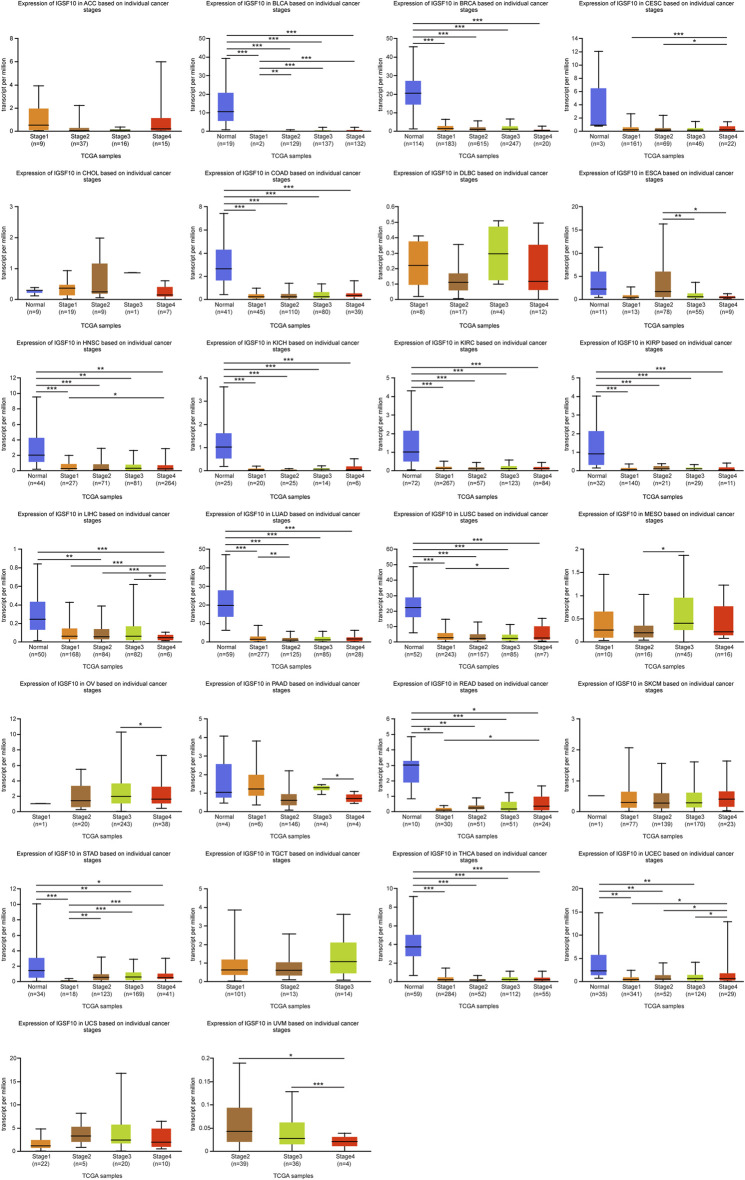
(Continued). Correlations between the *IGSF10* expression and tumor stage. ns, *p* ≥ 0.05, *p < 0.05, **p < 0.01, ***p < 0.001.

To study *IGSF10* expression levels across various tumor stages, we used the UALCAN online tool to compare *IGSF10* expression in tumor samples from patients at various tumor stages. We observed that the *IGSF10* was significantly reduced in the early tumor stages of 14 cancers (Figure 2), inclusive of BLCA, BRCA, COAD, HNSC, KICH, KIRC, KIRP, LIHC, LUAD, LUSC, READ, STAD, THCA, and UCEC, suggesting that *IGSF10* may have vital guiding magnitude for the early diagnosis of sufferers with these kinds of tumors.

### 3.2 Prognostic value of *IGSF10* in human pan-cancer

**FIGURE 3:**
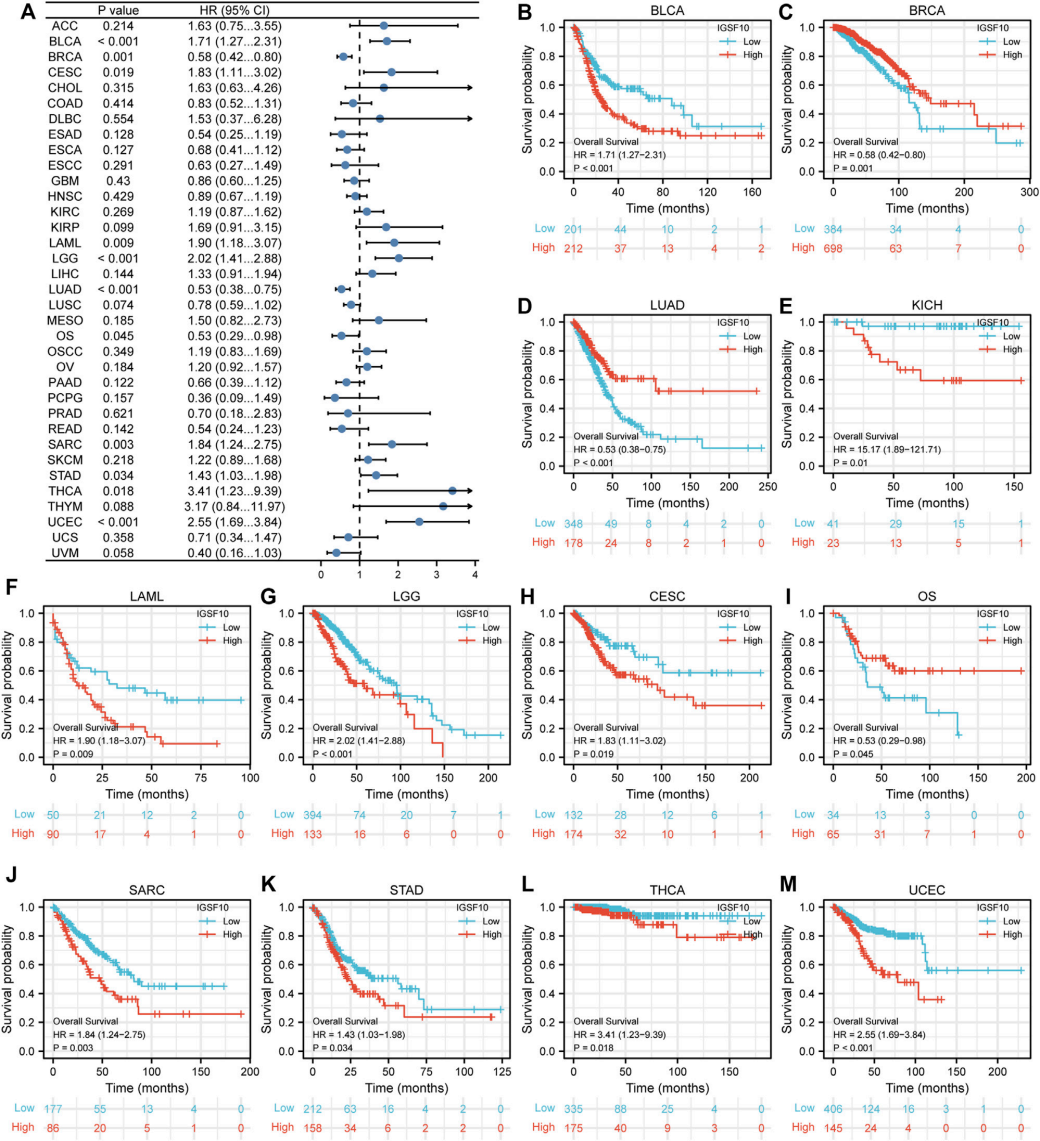
Correlations between the *IGSF10* expression and overall survival (OS). **(A)** Forest plot of results from the univariate survival analysis in pan-cancer for OS. **(B–M)** Kaplan-Meier survival curves showed associations between the *IGSF10* expression and OS.

To understand the prognostic value of *IGSF10* in a vary of cancers, we investigated correlations between *IGSF10* expression and OS of tumor sufferers by single variate Cox regression analysis. Our outcomes confirmed that *IGSF10* expression were drastically associated with OS in BLCA, BRCA, LUAD, KICH, LAML, LGG, CESC, Osteosarcoma (OS), SARC, STAD, THCA, and UCEC. Furthermore, *IGSF10* was a low-risk gene in BRCA, LUAD, and Osteosarcoma (OS), while it was a high-risk gene in BLCA, KICH, LAML, LGG, CESC, SARC, STAD, THCA, and UCEC (Figure 3).

**FIGURE 4:**
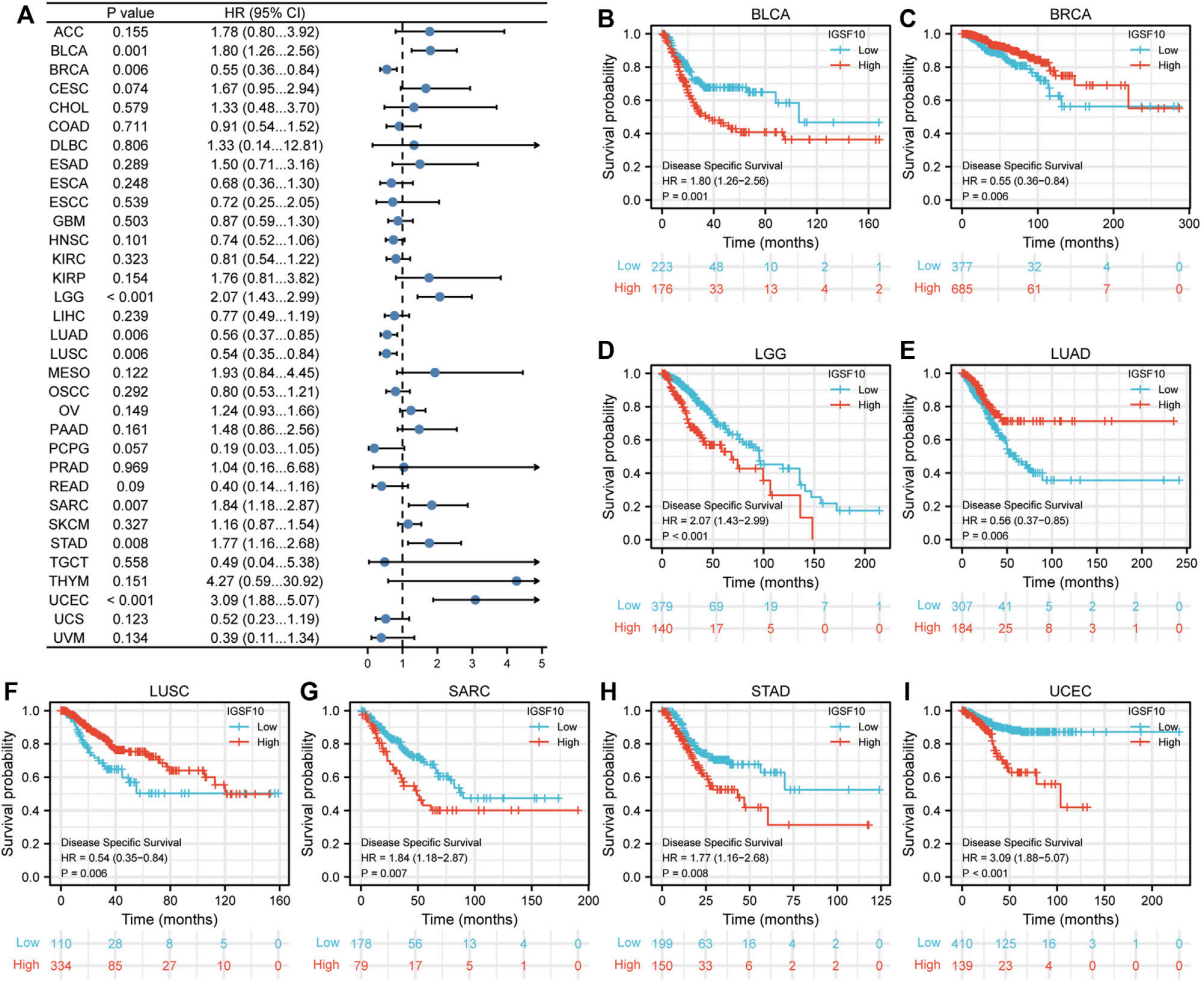
Correlations between the *IGSF10* expression and disease-specific survival (DSS). **(A)** Forest plot of results from the univariate survival analysis in pan-cancer for DSS. **(B–I)** Kaplan-Meier survival curves showed associations between the *IGSF10* expression and DSS.

Furthermore, we additionally examined correlations between *IGSF10* expression and DSS in a vary of cancers. Our findings confirmed that excessive *IGSF10* expression was a perilous factor for DSS in BLCA, LGG, SARC, STAD and UCEC. Interestingly, it was a beneficial factor in BRCA, LUAD and LUSC (Figure 4).

**FIGURE 5:**
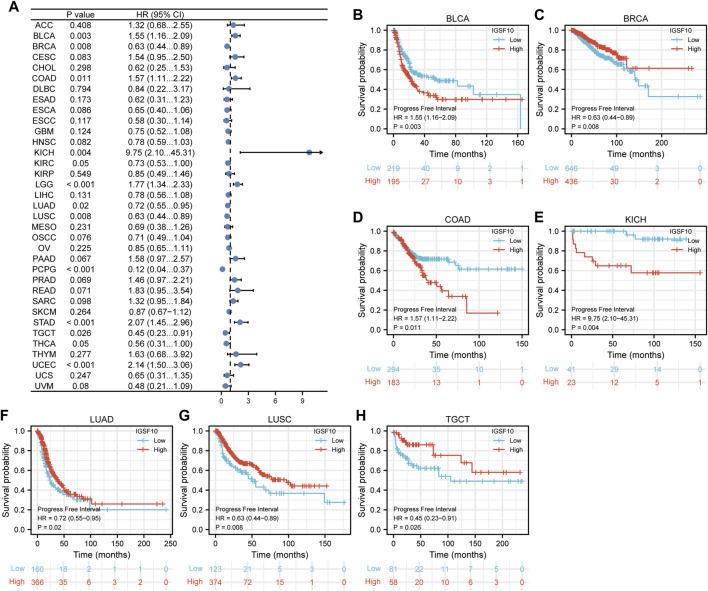
Correlations between the *IGSF10* expression and progression-free interval (PFI). **(A)** Forest plot of results from the univariate survival analysis in pan-cancer for PFI. **(B–H)** Kaplan-Meier survival curves showed associations between the *IGSF10* expression and PFI.

Next, correlations between *IGSF10* expression and PFI were also analyzed by Cox regression analysis. Our findings confirmed that excessive *IGSF10* expression was correlated with terrible PFI of cancer sufferers in BLCA, COAD and KICH, while low *IGSF10* expression was correlated with bad PFI of cancer sufferers in BRCA, LUAD, LUSC, and TGCT (Figure 5).

**FIGURE 6:**
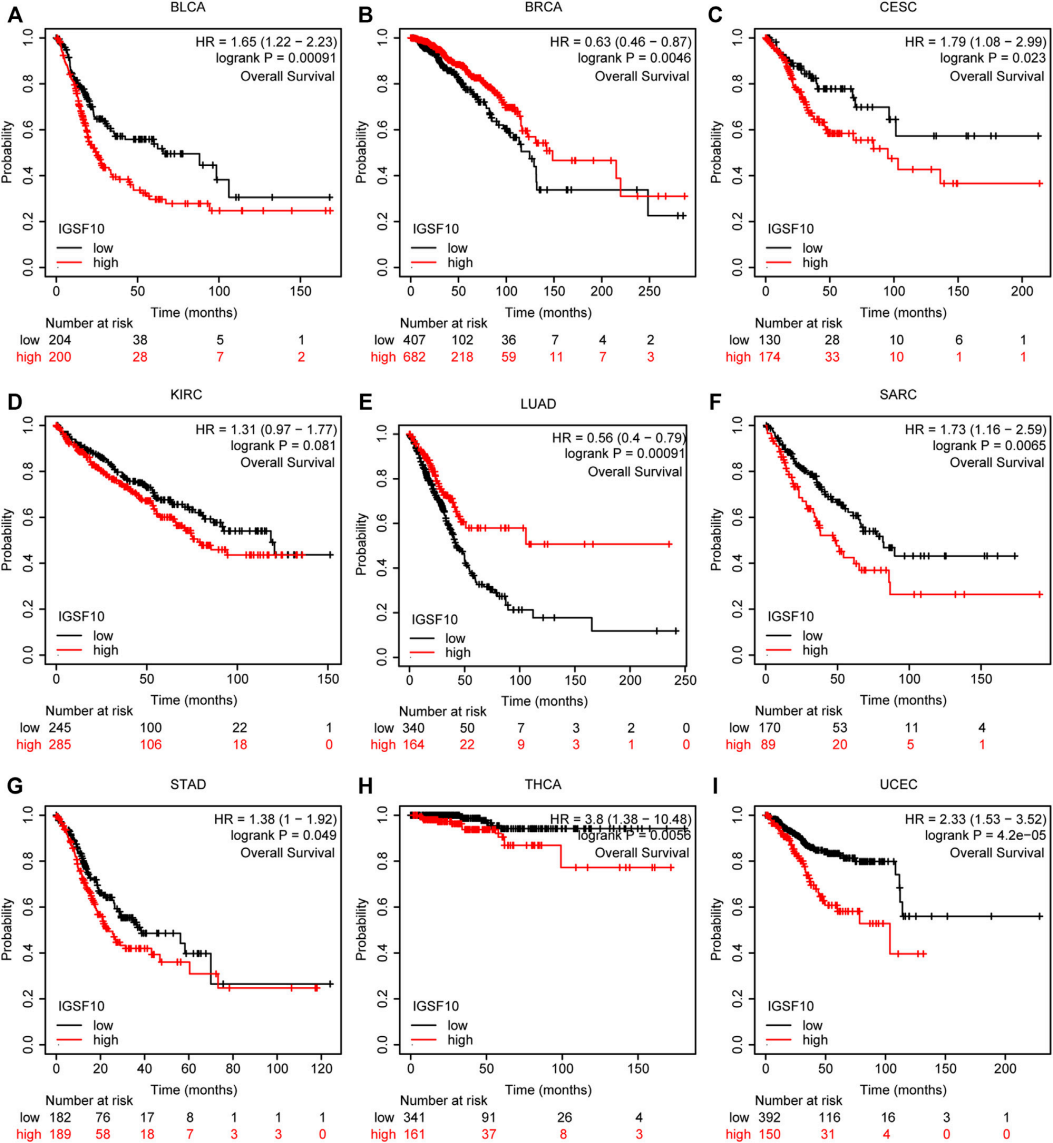
Associations between *IGSF10* expression and OS. **(A–I)** Correlations between the *IGSF10* expression and OS based on Kaplan-Meier Plotter database.

According to the Kaplan-Meier Plotter database, we further analyzed the correlations of *IGSF10* expression with OS. Our outcomes suggested that *IGSF10* expression performed a detrimental function in six kinds of cancer including BLCA, CESC, SARC, STAD, THCA, and UCEC. On the contrary, *IGSF10* expression owed a significant protective role in BRCA and LUAD (Figure 6). In addition, we also validated the relationships between *IGSF10* expression and prognosis using the GEO dataset. We found that *IGSF10* was a low-risk gene in BRCA, LUAD, and LUSC, while it was a high-risk gene in BLCA, Gastric cancer (GC), and Colorectal cancer (CRC) (Supplementary Figure S1).

Overall, the above consequences confirmed that *IGSF10* could function as a prognostic predictor for several types of cancer.

### 3.3 Correlations between *IGSF10* expression and immune infiltration in pan-cancer

**FIGURE 7:**
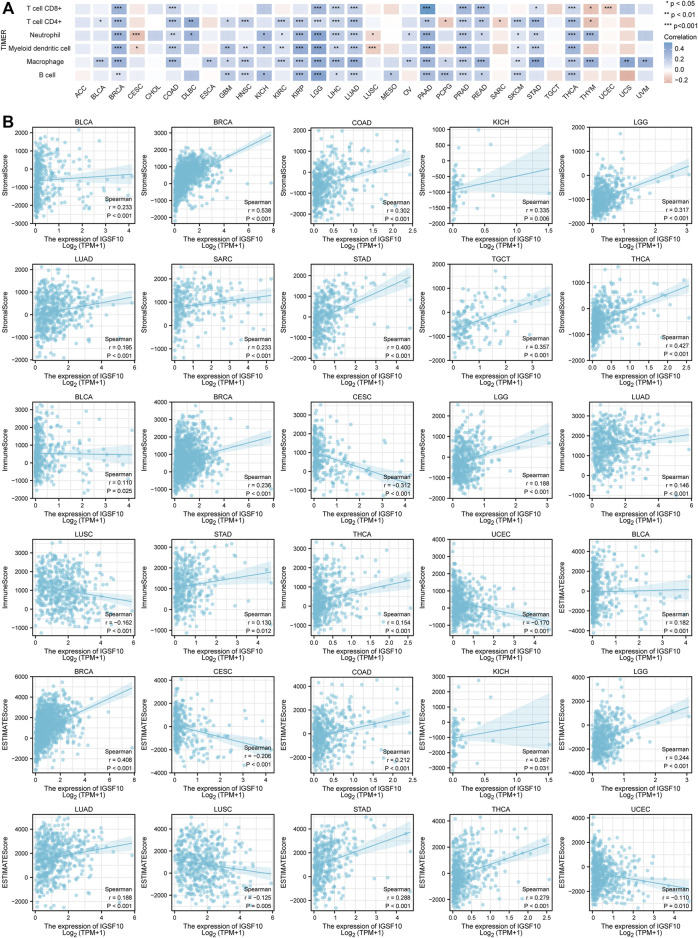
*IGSF10* expression was associated with cancer immunity. **(A)** Relationships between *IGSF10* expression and immune cell infiltration in different cancers. *p < 0.05, **p < 0.01, ***p < 0.001. **(B)** The correlations of *IGSF10* expression with the stromal score, immune score and ESTIMATE score in various cancers.

Immunotherapy has become another effective cancer treatment method besides chemotherapy, surgery, radiation therapy and targeted drug therapy (Murciano-Goroff et al., 2020). Notably development has been made in the application of immune checkpoint blockade in cancer therapy in latest years (Morad et al., 2021), but research showed that the proportion of sufferers who responded to checkpoint inhibitor drugs was estimated at 0.14% in 2011, and the percentage was estimated at 12.46% in 2018 (Haslam and Prasad, 2019). Based on this *status quo*, the search for novel predictive biomarkers is crucial to increase the proportion of individual patients who respond to immune checkpoint inhibitor. Therefore, we investigated the correlations between *IGSF10* expression and immune cell infiltration in a vary of cancers *via* the usage of the TIMER online database. The consequences confirmed that the *IGSF10* expression was notably related to CD8^+^ T cell infiltration in 12 kinds of tumors, CD4^+^ T cell infiltration in 20 tumors, neutrophils infiltration in 19 tumors, myeloid dendritic cells infiltration in 17 tumors, macrophages infiltration in 22 tumors and B cells infiltration in 15 tumors (Figure 7A). As *IGSF10* was found to show prognostic value in BLCA, BRCA, CESC, COAD, KICH, LAML, LGG, LUAD, LUSC, OS, SARC, STAD, TGCT, THCA, and UCEC, we examined stromal score, immune score and ESTIMATE score to evaluate the correlations between *IGSF10* expression levels and immune infiltration in a vary of cancers. The consequences confirmed that *IGSF10* expression was positively associated with the stromal score in BLCA, BRCA, COAD, KICH, LGG, LUAD, SARC, STAD, TGCT, and THCA. *IGSF10* expression was negatively associated with the immune score in CESC, LUSC, and UCEC. However, it was positively associated with the immune score in BLCA, BRCA, LGG, LUAD, STAD, and THCA. Furthermore, the *IGSF10* expression was notably negatively associated with ESTIMATE score in CESC, LUSC, and UCEC, while positively in BLCA, BRCA, COAD, KICH, LGG, LUAD, STAD, and THCA (Figure 7B).

### 3.4 Relationships between *IGSF10* expression and immune checkpoints, immune modulators

**FIGURE 8:**
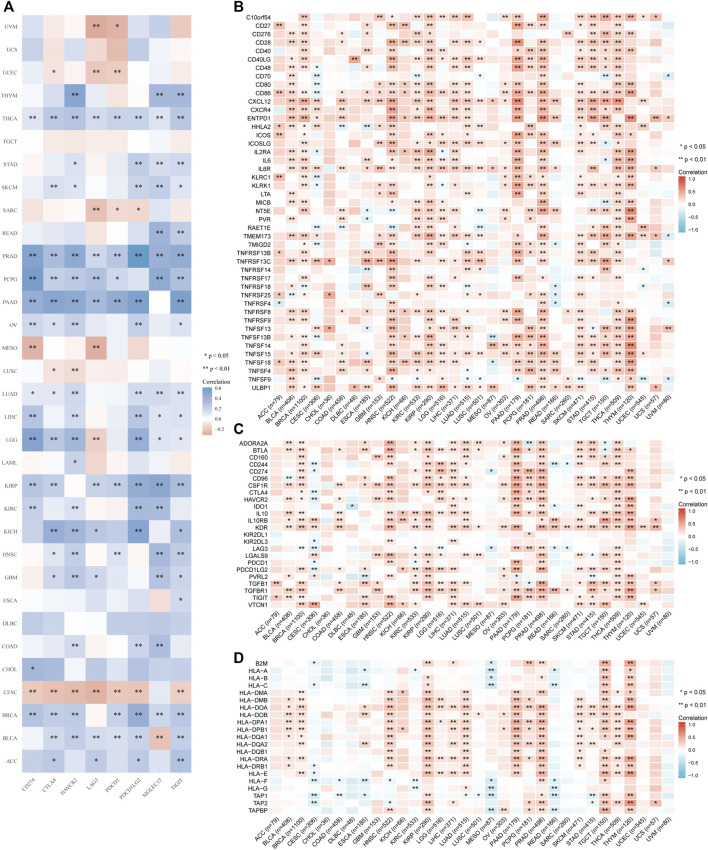
Correlation analysis of *IGSF10* and immune checkpoints, as well as immune modulators in pan-cancer. **(A)** The correlations of *IGSF10* and common immune checkpoints in pan-cancer. **(B)** The correlations of *IGSF10* and immune stimulators in pan-cancer. **(C)** The correlations of *IGSF10* and immune inhibitors in pan-cancer. **(D)** The correlations of *IGSF10* and MHC molecules in pan-cancer.

Furthermore, we analyzed correlations of *IGSF10* expression with common immune checkpoint genes. Interestingly, from the heatmap (Figure 8A), we can observe that nearly all immune checkpoint genes have been related to *IGSF10* in most cancer types, with the exception of CESC, LUSC, MESO, SARC, UCEC, and UVM, majority of immune checkpoint genes have been positively related to *IGSF10* in all kinds of tumors.

Besides, we investigated correlations between *IGSF10* expression and immune-related genes encoding immune stimulators, immune inhibitors, and MHC molecules in all kinds of tumors. The outcomes of the heatmap showed that nearly all immune-related genes have been positively associated with *IGSF10* in the vast majority of cancers (Figures 8B–D).

Altogether, the above data strongly support that *IGSF10* performs a crucial function in tumor immunity.

### 3.5 Correlation between *IGSF10* expression and TMB, MSI, MMR, and DNA methyltransferases in pan-cancer

**FIGURE 9:**
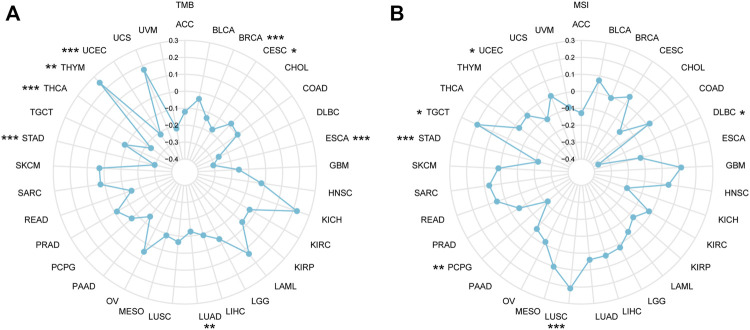
The correlations between *IGSF10* expression and TMB, MSI. **(A)** Radar map of the correlations between *IGSF10* expression and TMB in pan-cancer. **(B)** Radar map of the correlations between *IGSF10* expression and MSI in pan-cancer. *p < 0.05, **p < 0.01, ***p < 0.001.

TMB refers to the total quantity of genetic mutations assessed for a tumor specimen (Jardim et al., 2021). The conceptual definition of MSI is the hypermutator phenotype secondary to frequent polymorphism in short repetitive DNA sequences and single nucleotide substitution, resulting from MMR deficiency (Baretti and Le, 2018). In recent years, increasing evidence indicated that TMB and MSI function as biomarkers for predicting the immunotherapy response (Chan et al., 2019; Liu et al., 2019; Samstein et al., 2019; Yamamoto and Imai, 2019; Chen, 2022), so we examined correlations between *IGSF10* and TMB, MSI. As proven in Figure 9A, *IGSF10* expression was positively associated with TMB in THYM, and negatively associated in BRCA, CESC, ESCA, LUAD, STAD, THCA, and UCEC. The expression of *IGSF10* was positively associated with MSI in LUSC and TGCT, however negatively associated in DLBC, PCPG, STAD, and UCEC (Figure 9B).

**FIGURE 10:**
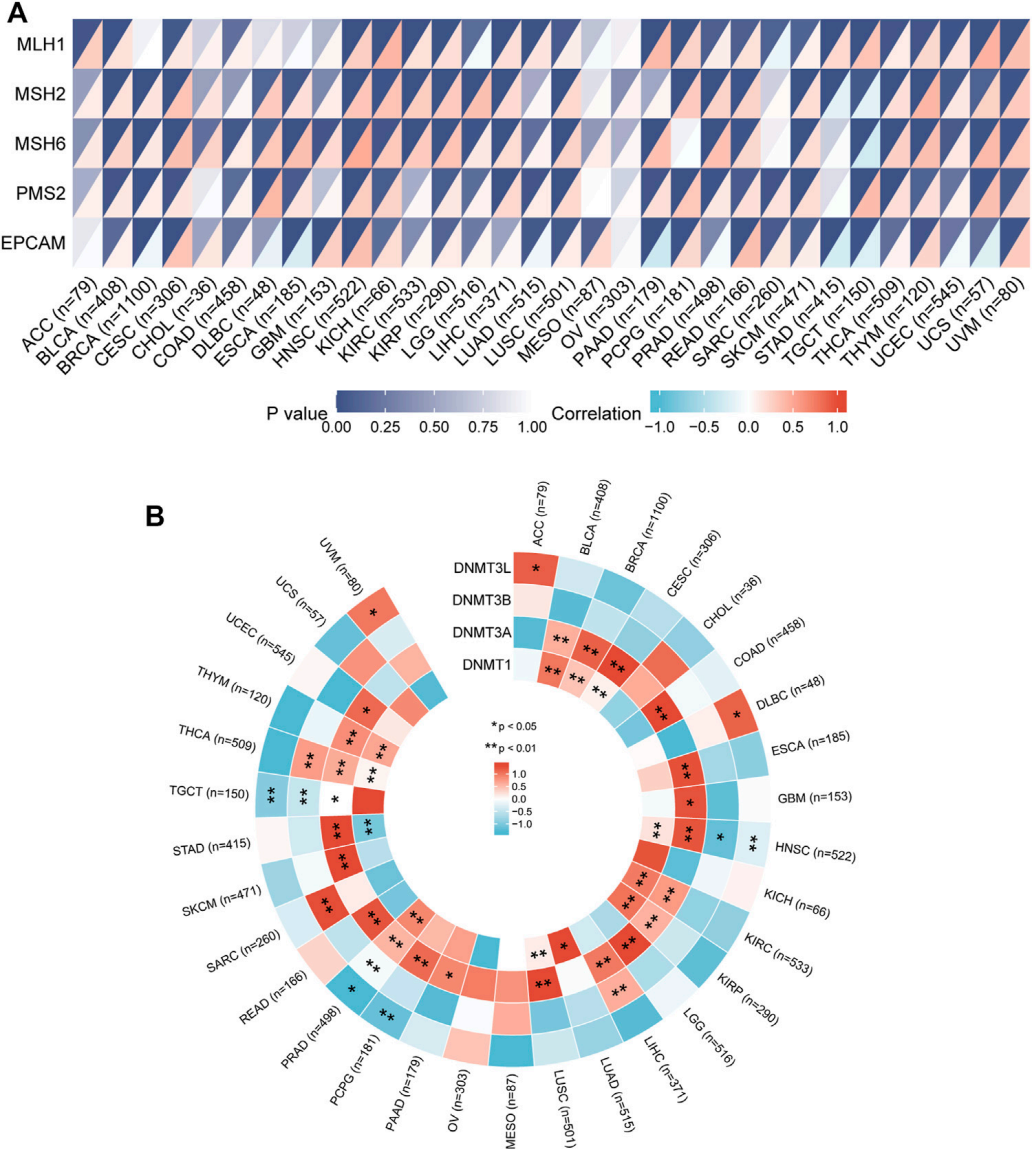
The associations of *IGSF10* expression with MMR genes and DNA methyltransferases in pan-cancer. **(A)** Correlations between *IGSF10* expression and fiveMMR genes (*MLH1*, *MSH2*, *MSH6*, *PMS2*, *EPCAM*) expression. The lower right triangle of each cell represents the correlation coefficient calculated by Spearman’s correlation test, and the upper left triangle represents the *p*-value. **(B)** Correlations between *IGSF10* expression and four DNA methyltransferase genes. *p < 0.05, **p < 0.01

MMR is a security system in cells and an evolutionarily highly conserved biological process responsible for repairing base mismatches that occur during DNA replication (Hermans et al., 2016). Dysregulation of the MMR system can alter cellular biological functions, thereby promoting tumorigenesis or promoting the malignant progression of tumors (Germano et al., 2017). To explore the potential function of *IGSF10* in tumorigenesis and development, we assessed associations of *IGSF10* expression with mutation levels of five MMR genes. The outcomes confirmed that *IGSF10* was noticeably positively associated with MMR genes in 27 kinds of cancers, besides CHOL, MESO, OV, STAD and TGCT (Figure 10A). We also examined relationships between *IGSF10* and four DNA methyltransferases. The expression of *IGSF10* was highly positively related to these four DNA methyltransferases in 24 cancers, except CHOL, KICH, MESO, OV, PCPG, STAD, TGCT, and UCS (Figure 10B). These consequences suggest that *IGSF10* may affect tumorigenesis and development by means of regulating DNA repair and DNA methylation in cancers.

### 3.6 Widespread genetic alterations of *IGSF10* in pan-cancer

**FIGURE 11:**
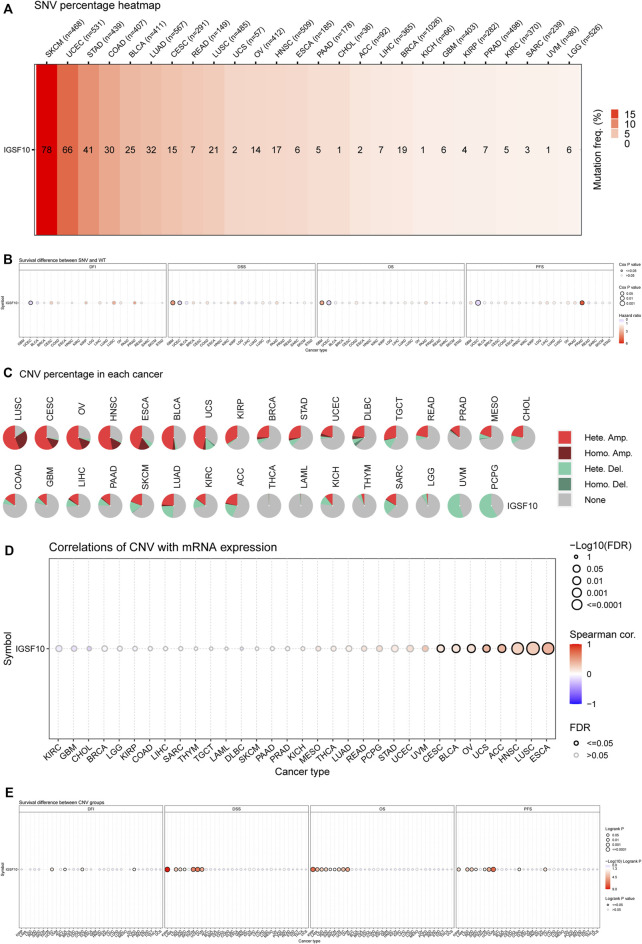
(Continued). Genetic alterations of *IGSF10* across different cancers from the GSCA database. **(A)** The profile of SNV of *IGSF10* gene set in pan-cancer. **(B)** Bubble plot of the survival difference between mutant (deleterious) and wide type in pan-cancer. **(C)** Pie plot summarizes the CNV of *IGSF10* gene in pan-cancer. **(D)** The correlations between CNV and *IGSF10* mRNA expression in pan-cancer. **(E)** The difference of survival between CNV and wide type in pan-cancer.

Firstly, the SNV information of 10,234 samples from all kinds of cancers have been gathered from TCGA database. As shown in Figure 11A, the percentage heatmap summarized the frequency of deleterious mutations in pan-cancer. SNV was found in 26 cancer types and mutations were most common in SKCM and UCEC. Furthermore, SNV of *IGSF10* was related to a significantly beneficial prognosis for DFI in UCEC. SNV of *IGSF10* indicated a significantly poor prognosis for DSS and OS in GBM, but the opposite trend in UCEC. The SNV of *IGSF10* in PRAD indicated a significantly poor prognosis for PFS, whereas the opposite was found in UCEC (Figure 11B). We also analyzed the CNV of *IGSF10* and the correlation of CNV with mRNA expression through the GSCA database (Figures 11C, D). CNV pie plot represented the proportion of different types of CNV of *IGSF10* gene in each cancer. Bubble plot confirmed that CNV of *IGSF10* was positively related to mRNA expression in eight cancers, including CESC, BLCA, OV, UCS, ACC, HNSC, LUSC, and ESCA. Likewise, we investigated the impact of CNV of *IGSF10* on the prognosis of patients with various cancers. In UCEC, BLCA, COAD, PCPG, KIRP, LAML, LGG, PAAD, SARC, SKCM, THYM, UVM, and KIRC, the CNV of *IGSF10* was associated with prognosis (Figure 11E).

**FIGURE 12:**
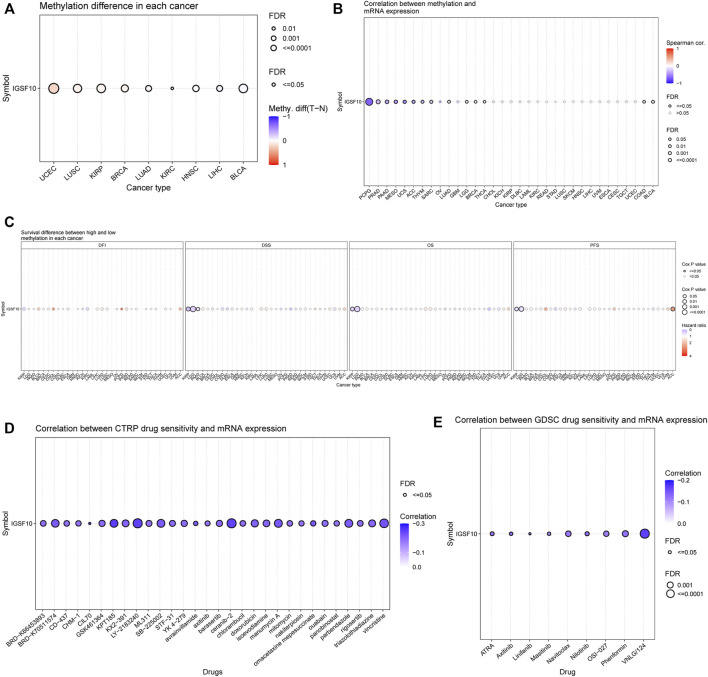
DNA methylation aberration of *IGSF10* across different cancers and correlation of *IGSF10* expression with drug (top 30) sensitivity from the GSCA database. **(A)** The methylation difference between tumor and normal samples of *IGSF10* gene in pan-cancer. **(B)** The profile of correlations between methylation and mRNA expression of *IGSF10* gene in pan-cancer. **(C)** The overall survival difference between higher and lower methylation groups in pan-cancer. **(D)** The correlations between *IGSF10* gene expression and the sensitivity of CTRP drugs (top 30) in pan-cancer. **(E)** The correlations between *IGSF10* gene expression and the sensitivity of GDSC drugs (top 30) in pan-cancer.

Methylation of *IGSF10* in a vary of cancers was analyzed *via* the usage of GSCA database. Bubble chart showed differences between *IGSF10* gene methylation levels in various tumor and normal specimens. We found that *IGSF10* was significantly hypermethylated in UCEC, LUSC, KIRP, BRCA, LUAD, and KIRC samples compared with that in normal samples (Figure 12A). Figure 12B showed the correlation results between *IGSF10* mRNA expression and *IGSF10* methylation levels in various tumors. Methylation of *IGSF10* was negatively correlated with *IGSF10* mRNA expression in PCPG, PRAD, PAAD, MESO, and UCS etc. The association between methylation of *IGSF10* and prognosis in pan-cancer was further analyzed. As shown in Figure 12C, patients with hypermethylation of *IGSF10* had a good prognosis in KIRP, LGG and PAAD, but the opposite trend in ACC.

### 3.7 The correlation between *IGSF10* expression and the sensitivity of small-molecule drugs in pan-cancer

To explore whether the *IGSF10* expression was correlated with drug sensitivity, Pearson correlation analysis was used to detect correlations between *IGSF10* expression and the drug sensitivity (IC50) of various anticancer drugs in the GDSC and CTRP databases. The drug sensitivity of almost all drugs shown in Figures 12D, E was negatively associated with *IGSF10* mRNA expression. These data strongly advised that *IGSF10* could act as a biomarker for predicting drug response.

### 3.8 Enrichment of *IGSF10*-related partners

**FIGURE 13:**
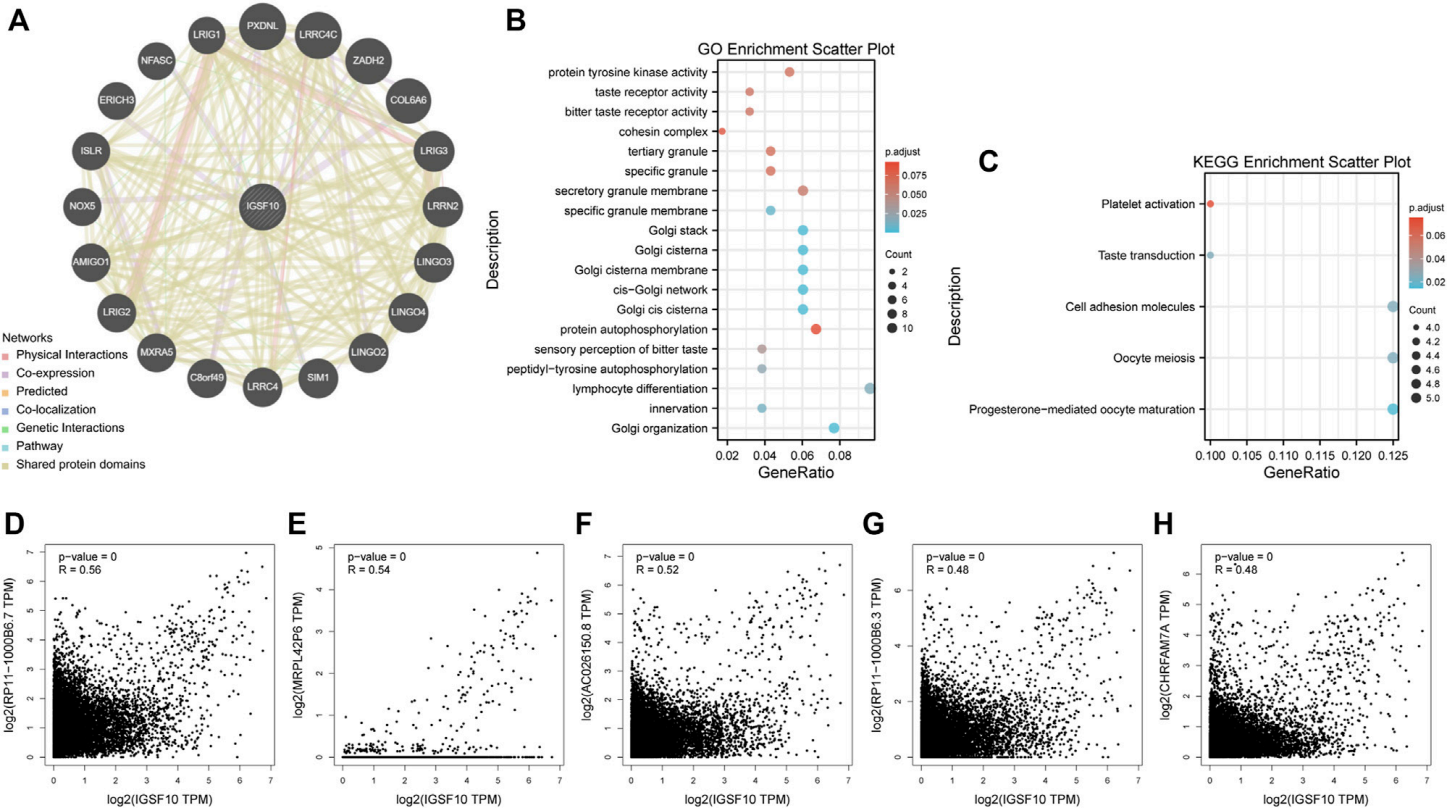
Enrichment analysis of IGSF10-interacted proteins and *IGSF10*-related genes. **(A)** IGSF10-interacted proteins from the GeneMANIA database. **(B)** GO enrichment analysis based on the IGSF10-interacted proteins and *IGSF10*-related genes. **(C)** KEGG enrichment analysis based on the IGSF10-interacted proteins and *IGSF10*-related genes. **(D–H)** Scatter plots of expression correlations between *IGSF10* and *RP11-1000B6.7*, *MRPL42P6*, *AC026150.8*, *RP11-1000B6.3,* and *CHRFAM7A* in pan-cancer using the GEPIA2 database.

To reveal molecular mechanism of *IGSF10* in tumorigenesis and progression, we integrated *IGSF10*-related genes and IGSF10-binding proteins for enrichment analysis. Firstly, we obtained 20 IGSF10-binding proteins through the GeneMANIA database (Figure 13A). In addition, we got the top 1000 *IGSF10* expression-related genes through GEPIA2 database. *IGSF10* expression was positively related to the expression of the top five genes, including *RP11-1000B6.7* (R = 0.56), *MRPL42P6* (R = 0.54), *AC026150.8* (R = 0.52), *RP11-1000B6.3* (R = 0.48) and *CHRFAM7A* (R = 0.48) (Figures 13D–H). Furthermore, KEGG and GO enrichment analyses have been carried out on these IGSF10-binding proteins and *IGSF10*-related genes. The GO enrichment results showed that most of IGSF10-binding proteins and *IGSF10*-related genes were related to lymphocyte differentiation, Golgi organization, Golgi cis cisterna, and others (Figure 13B). The KEGG results further suggested that IGSF10 could adjust the occurrence and development of cancers *via* participating in the “cell molecules adhesion” signaling pathway (Figure 13C).

## 4 Discussion

*Cancer* is caused by a variety of factors, mainly including exogenous and endogenous factors (Neveu et al., 2020). With the advent of genetic testing and the era of targeted therapy, molecular signatures have become increasingly critical, which can predict individual patients prognosis or predict individual patients response to specific treatments (Michiels et al., 2016). Hence, there is a pressing want to discover effective biomarkers to precisely assess the prognosis and effectively improve the treatment of tumor patients.

Our study aimed to comprehensively analyze function of *IGSF10* in all kinds of tumors. We obtained expression data of *IGSF10* in various cancers from multiple friendly public databases open to the world, which was helpful to discover variations in *IGSF10* expression in a vary of cancers. Our findings indicated that *IGSF10* expression were notably different between tumor and normal tissues across multiple cancer types. Among them, *IGSF10* expression levels were downregulated in ACC, BLCA, BRCA, CESC, COAD, ESCA, HNSC, KICH, KIRC, KIRP, LIHC, LUAD, LUSC, OV, PRAD, READ, SKCM, STAD, TGCT, THCA, and UCEC, but the opposite trend in DLBC, GBM, LAML, LGG, PAAD, and THYM. Furthermore, we investigated the prognostic function of *IGSF10* in multiple cancers *via* the usage of three prognostic indicators: OS, DSS and PFI. On the basis of our results, we found that *IGSF10* played a beneficial role in five tumors including BRCA, LUAD, LUSC, Osteosarcoma (OS) and TGCT. In contrast, *IGSF10* expression owed a significant detrimental role in BLCA, CESC, COAD, KICH, LAML, LGG, SARC, STAD, THCA, and UCEC.

Multiple researches have showed that infiltrating immune cells in tumor specimens influenced malignant tumor development (Fridman et al., 2012; Garnelo et al., 2017; Kalafati et al., 2020). More recently, a growing quantity of researches have revealed that tumor-infiltrating immune cells were closely correlated with the prognosis of tumor patients (Gooden et al., 2011; Loi et al., 2014; Schalper et al., 2015; Goode et al., 2017; Disis et al., 2019; Stewart et al., 2019). Our research showed that IGSF10 may perform a specific function in regulating tumor immunity. In our research, we provided evidence of the relationships between *IGSF10* expression and immune infiltration, immune checkpoints, immune modulators, etc. in multiple tumors. We observed notably correlations between *IGSF10* expression and infiltration levels of CD8^+^ T cells, CD4^+^ T cells, neutrophils, myeloid dendritic cells, macrophages and B cells in most cancer types. Furthermore, ESTIMATE was described as a method for estimating the proportion of stromal and immune cells in tumor specimens *via* the usage of gene expression signatures (Yoshihara et al., 2013). It is worth noting that *IGSF10* expression was significantly negatively related to ESTIMATE score in CESC, LUSC, and UCEC, while positively in BLCA, BRCA, COAD, KICH, LGG, LUAD, STAD, and THCA. Our results also showed that *IGSF10* was associated with almost all immune checkpoint genes in most types of tumors, *IGSF10* was positively associated with majority of immune checkpoint genes in all kinds of tumors. Additionally, our study demonstrated that close to all immune-related genes were positively associated with *IGSF10* expression in vast majority of tumors. These outcomes all confirmed that *IGSF10* expression was closely correlated with the tumor-infiltrating immune cells, affected the prognosis of sufferers, and provided a novel target for the development of immunosuppressants.

TMB is an emerging good biomarker that has recently attracted widespread attention to predict response of individual patients to immune checkpoint inhibitor (ICI) (Samstein et al., 2019; Fumet et al., 2020; Sampson et al., 2020), which could lead immuno-oncology rapidly into the period of precision medicine (Steuer and Ramalingam, 2018). MSI is characterized by high-frequency frameshift mutations in microsatellite DNA, caused by MMR deficiency that fails to repair insertion or deletion mutations during DNA replication (Pal et al., 2008; Song et al., 2018; El-Deiry et al., 2019; McGrail et al., 2020). MMR deficiency causes both MSI and high TMB(Georgiadis et al., 2019; Willis et al., 2020). Further, MMR and MSI also may predict clinical response to ICI(Hodges et al., 2017; Le et al., 2017; Janjigian et al., 2018; Cortez, 2019). The outcomes of our research showed that *IGSF10* expression was related to TMB in BRCA, CESC, ESCA, LUAD, STAD, THCA, THYM, and UCEC. Our findings also showed that *IGSF10* expression was related to MSI in DLBC, LUSC, PCPG, STAD, TGCT, and UCEC. In addition, our research confirmed that *IGSF10* was noticeably positively related to MMR genes in 27 kinds of tumors, besides CHOL, MESO, OV, STAD, and TGCT. Taken together, the above outcomes suggested that *IGSF10* expression may affect the TMB, MSI and MMR of tumors, thereby exerting influence on the response of individual patients to ICI therapy. The above findings suggested that IGSF10 was expected to provide a valuable reference for predicting response of individual patients to immunotherapy. DNA methylation is a common, stably inherited epigenetic modification, and it can influence gene regulation (Catoni et al., 2018). Previous researches have proven that aberrant methylation is relate to tumorigenesis and immune evasion of tumors (Cheng et al., 2015; Jung et al., 2019; Zhao et al., 2020). The *IGSF10* expression was noticeably positively correlated with these four DNA methyltransferases in 24 cancers, except CHOL, KICH, MESO, OV, PCPG, STAD, TGCT, and UCS. These outcomes propose that aberrant *IGSF10* expression may play a critical function in tumorigenesis by regulating DNA methylation in cancers.

The genetic variations include CNVs and SNVs, which can be inherited or *de novo* (Mercati et al., 2017). The accumulation of SNV performs a very important function in tumorigenesis and tumor progression (Abelson et al., 2020). In addition to SNVs, DNA copy number alterations and chromosomal instability are a hallmark of cancer (Seifert et al., 2016). To date, few studies have reported on relationships between *IGSF10* gene variations and cancers. In this study, we found that *IGSF10* has different degrees of SNVs and CNVs in most cancer types, among which *IGSF10* is most prone to SNVs in SKCM and UCEC. Furthermore, we found that *IGSF10* mutations were significantly correlated with prognosis of patients in multiple tumor types.

In addition, we combined IGSF10-interacted proteins and *IGSF10*-related genes for enrichment analysis. Gene enrichment analysis revealed that IGSF10 may have an effect on the occurrence, progression or immunity of cancer *via* participating in lymphocyte differentiation and cell molecules adhesion.

## 5 Conclusion

In summary, our systemic pan-cancer analysis showed for the first time aberrant *IGSF10* expression across different tumors. Furthermore, we found that *IGSF10* can serve as a valuable prognostic biomarker for certain types of cancer. According to our findings, the level of *IGSF10* is associated with cancer immunity, providing a new idea for individualized cancer immunotherapy, and is expected to be a potential immunological and prognostic biomarker.

## Data Availability

The original contributions presented in the study are included in the article/Supplementary Material, further inquiries can be directed to the corresponding author.

## Supplementary material

SUPPLEMENTARY FIGURE S1 Associations between *IGSF10* expression and prognosis. **(A–L)** Correlations between the *IGSF10* expression and prognosis based on the GEO dataset.
