# Supplementary figures and images for: Pan-cancer analyses reveal IGSF10 as an immunological and prognostic biomarker
Source: Front Genet. 2023 Jan 4;13:1032382. doi: 10.3389/fgene.2022.1032382 (PMC9845414; doi:10.3389/fgene.2022.1032382)

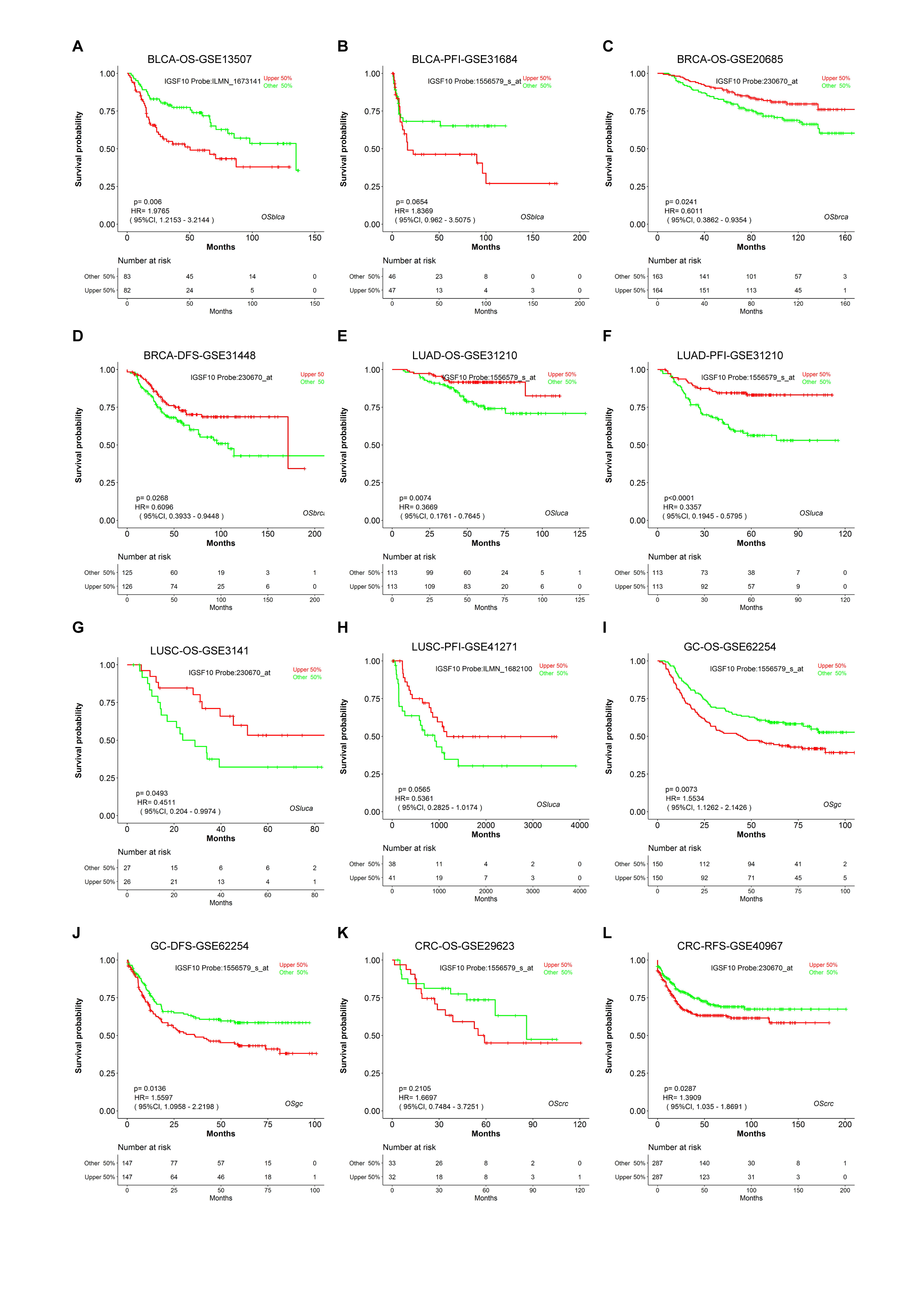

Supplement: Supplementary file 1 [file Image1.TIF]
